# Tilting of the Cardiac Axis During Dobutamine Stress Echocardiography: Potential Marker for Ischemia

**DOI:** 10.7759/cureus.15605

**Published:** 2021-06-11

**Authors:** Preetham Gunta, Angel López-Candales, Paramdeep Baweja, Michael Sweeney

**Affiliations:** 1 Internal Medicine, University of Missouri Kansas City, Kansas City, USA; 2 Cardiovascular Medicine, University of Missouri Kansas City, Kansas City, USA

**Keywords:** cardiac axis, cardiac axis tilt, ischemic heart disease, transthoracic echocardiogram, cardiac stress test

## Abstract

Stress echocardiography is a tool for assessing the extent and severity of coronary artery disease (CAD) during physical or pharmacological stress. Transient worsening of regional left ventricular (LV) function during stress is a well-recognized abnormality of inducible ischemia.

We present the case of a 57-year-old female with risk factors for CAD who was referred for a dobutamine stress echocardiogram for complaints of typical angina. It was interpreted as positive for inducible ischemia, but using unconventional criteria. Unfortunately, this study had reduced sensitivity due to LV hypertrophy. All LV wall segments were not clearly seen to comment on regional contractility, and an abnormal cardiac tilt from its axis was noted, suggestive of ischemia along the anteroseptal, anterior and lateral walls. Following this, a coronary angiogram showed diffuse CAD.

The cardiac axis with the presence of a tilt as a potential measure of ischemia is previously unrecognized. The idea invokes a mathematical principle based on the direction and the magnitude of the vector of opposing walls during contractility. Simply implying that ischemic segments might contract in the same direction, vector magnitude will be less prominent; hence, “axial tilt” will occur. Prospective studies are needed to validate the feasibility and reproducibility of this abnormality in the assessment of ischemia and its viability in clinical practice.

## Introduction

Stress test is an excellent noninvasive means to better estimate the pretest probability of patients undergoing a more definitive test to establish coronary artery disease (CAD), that is, coronary angiography. Of the many variants of this test, a very popular one is dobutamine stress echocardiography (DSE). While we can obtain significant information through a DSE, it remains a challenging test to perform for the technician and still is largely interpreter-dependent. One cannot overstate the importance of an intermediate step between mild suspicion for CAD and coronary angiography in the appropriate situation, especially considering the adverse health consequences of the latter. Considering this, any additional finding that can help us rule in or rule out demonstrable ischemia would be invaluable. We present one such phenomenon through this case.

## Case presentation

We present the case of a 57-year-old Caucasian female who was referred to the cardiology clinic for complaints of chest pain. The chest pain was central and dull in nature and aggravated with even minimal exertion like washing dishes and alleviated with rest. This was accompanied by palpitations and diaphoresis. Aside from these complaints, she also noted shortness of breath on walking just one flight of stairs, decreased exercise tolerance, three-pillow orthopnea, and bilateral leg swelling. She had a history of hypertension, moderately well controlled on carvedilol, lisinopril, and spironolactone. The patient also had a 30-pack-year smoking history. The patient’s mother was 61 when she died of an unknown heart disease. By estimation, her 10-year cardiovascular event risk was 12.1%. Further workup was pursued to investigate for an obstructive CAD as the possible etiology.

A standard DSE was performed with the addition of atropine and the target heart rate was reached. Unfortunately, peak stress image acquisition was somewhat challenging, and even though all left ventricular (LV) wall segments were not clearly seen to comment on regional contractility, an abnormal cardiac tilt from its axis was noted from the four- and two-chamber views. This was suggestive of the presence of ischemia along the anteroseptal, anterior and lateral walls. The presence of LV hypertrophy and poor image quality reduced the overall test sensitivity to fully assess the extent of the abnormality. Following this, a coronary angiogram showed diffuse 70% disease in the left main, 70% ostial left anterior descending, and 90% ostial left circumflex with 70% lesion of the right coronary artery. As there were left main disease and three-vessel disease, the patient was scheduled to consult the cardiothoracic surgery team for coronary artery bypass graft.

**Figure 1:**
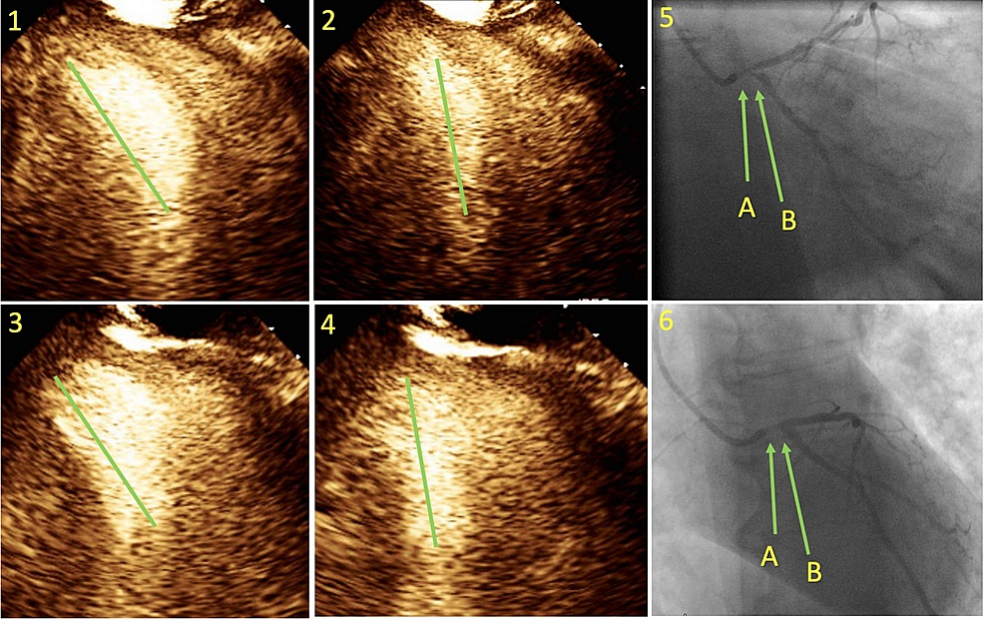
Dobutamine stress echocardiography and coronary angiogram images. Images 1 and 2 are two-chamber views of the heart in diastole and systole exhibiting tilt of the cardiac axis. Images 3 and 4 similarly represent the heart in four-chamber views. The green vertical lines in images 1, 2, 3, and 4 represent the imaginary line that passes through the center of the left ventricular cavity, i.e., the cardiac axis. Images 5A and 6A show severe left main stenosis with pressure dampening without contrast reflux. Images 5B and 6B show severely stenotic ostium of the left circumflex artery.

## Discussion

LV function is largely dependent on a series of complex interactions not only involving myocardial tissue architecture and contractility but also loading conditions [1]. It has been demonstrated that myocardial fibers within the LV wall follow a specific double-helical arrangement [2]. Specifically, LV myofibers in the myocardial wall gradually change from a right-handed helix in the sub-endocardium to a left-handed helix in the sub-epicardium, being almost horizontal in the mid-wall [3]. The helical arrangement of these LV myofibers is responsible for the sliding or shear deformation resulting in LV counterclockwise rotation of the apex and a clockwise rotation of the LV base [4]. The resultant twist not only contributes to maintaining a uniform distribution of LV fiber stress and fiber shortening across the LV wall but also produces a relatively high ejection fraction despite limited myofiber shortening [4-8].

LV twisting and shearing of the sub-endocardial fibers during ejection result in storage of potential energy, which is subsequently used for diastolic uncoiling of circumferential fibers with counterclockwise uncoiling of the LV fibers and untwisting of helices to produce diastolic suction [9]. Therefore, the LV twist provides a key mechanistic link between systole and diastole. When seen from standard four-chamber apical window views during echocardiographic imaging, LV contractility can be appreciated not only as a reduction in systolic volumes but also as longitudinal basal to apical displacement of the lateral mitral annulus [10]. In the absence of any clinical entity that affects LV-RV interdependence such as constriction, severe lung disease, and pulmonary hypertension, interventricular septal contractility abnormalities occur after cardiac surgery due to pacing or other conduction abnormality or due to eccentric cardiac remodeling among others. Tilt from the apical axis does not occur and the cycle of LV contractility-relaxation maintains a rather perpendicular orientation without a shift from this perpendicular axis with no appreciable tilt. The latter can be mechanistically explained based on simple vector principles. If both the magnitude and direction of the two vectors are equal based on the same wall thickness and effective contractility, both inferoseptal and lateral walls will be seen to contract, and its imaginary vertical perpendicular axis drawn from the middle of the LV cavity, as seen for the four-chamber apical window, will not tilt from its axis. In a similar fashion, if both inferior and anterior walls are normal, as seen from the two-chamber apical view, their contractility would not result in a tilt if an imaginary line is also drawn from the mid-LV cavity towards the apex. However, if one of the opposite walls is hypokinetic in relation to the other wall, this will certainly compromise the balance of vector forces between the two walls resulting in a tilt from the axis favoring the more contractile and dynamic wall. Thus, as the degree of hypokinesis increases, we would expect to see a greater tilt degree in the opposite direction of the compromised wall. The latter would explain our stress echo findings.

We did find mention of cardiac axis in maternal-fetal medicine literature, but it was vastly different in definition and clinical significance. The fetal cardiac axis has been defined as a line passing through the interventricular septum in a four-chamber view. This is commonly used to determine the fetal heart position; The normal fetal cardiac axis is said to lie approximately 45 degrees to the left of the anteroposterior line passing from the spine to the sternum [11,12]. This is different from our definition, as mentioned above or as shown below. Clinically, an abnormal fetal cardiac axis/heart position is an indication to further explore the possibility of an intrathoracic anomaly. We propose that our definition be used only in adults. This is because the tilt in the cardiac axis is a subtle parameter to observe and can be difficult with fetal hearts due to excessive cardiac motion and faster heart rates. Further, we intend to use it to predict an ischemic state which is extremely rare in a fetal heart.

**Video 1:** Animation demonstrating the abnormal cardiac axis tilt. We assume the dotted line to be the cardiac axis which is a straight line passing through the apex and the mitral valve. On the right, normal wall motion, without any tilt of the axis; in the middle, inferior wall motion is hypokinetic with an axis tilt towards the anterior wall; on the left, the inferior wall is akinetic with a more apparent axis tilt towards the anterior wall.

## Conclusions

Based on these principles, if there is an abnormal tilt of the cardiac axis away from a particular wall of the LV, it can be inferred that (i) the vectors of the opposing walls are asymmetric, (ii) one of the two walls (in some cases, both) is (are) hypokinetic/akinetic, and (iii) the wall from which the axis is moving away is the hypo/akinetic wall. By virtue of this, ischemia and coronary disease are implied. Unfortunately, lack of imaging standardization would preclude routinely using this tilting principle in standard stress echocardiography imaging unless we apply this principle to speckle tracking imaging so that specific landscape markings can be used for this purpose.
